# Novel lacto-peptides improve cognitive decline

**DOI:** 10.18632/aging.101893

**Published:** 2019-03-28

**Authors:** Yasuhisa Ano, Hiroyuki Nakayama

**Affiliations:** 1 Research Laboratories for Health Science & Food Technologies, Kirin Company Ltd, Yokohama, Kanagawa, Japan; 2 Graduate School of Agricultural and Life Sciences, the University of Tokyo, Bunkyo-ku, Tokyo, Japan

**Keywords:** dairy product, dopamine, memory, peptide, whey

Given the rapid growth in aging populations worldwide, the prevention of age-related memory decline and dementia has become a high priority. Several epidemiological and clinical studies have suggested that fermented dairy products help the prevention of cognitive decline [1,2]; specifically, the intake of Camembert cheese, a dairy product fermented with fungi, was shown to prevent Alzheimer’s pathology in mouse models [3]. However, the responsible ingredients for this prevention in fermented dairy products remain unclear. To elucidate the molecular mechanisms underlying the preventive effects of fermented dairy products on cognitive decline, we screened various peptides from digested fermented dairy products for their ability to improve memory impairment using a scopolamine-induced amnesia mouse model [4].

The effects of a single administration of whey protein digestions with various enzymes were evaluated. Amnesia mouse models injected with scopolamine were orally administered whey digestions and subjected to the Y-maze test at 1 hour later. Spontaneous alternations in this test were increased in mice administered with whey digestions by proteases from *Aspergillus melleus* or *Bacillus stearothermophilus.* Through the repeated fractionations of the whey digestions and behavioral evaluations, we identified glycine-threonine-tryptophan-tyrosine (GTWY) peptide, as an ingredient responsible for improving memory impairment. GYWY peptide not only improved short-term spatial working memory in scopolamine-induced amnesia mice, but also enhanced long-term object recognition memory in normal mice. Moreover, the consumption of GTWY peptide or whey peptide rich in GTWY peptide improved age-related cognitive decline in aged mice.

To elucidate molecular mechanisms of the memory improvement by GTWY peptide, we investigated its kinetics and involvement in monoamine productions. Orally administered ^14^C-GTWY peptide was smoothly moved into the blood, and the ratio of radioactivity in the hippocampus and cerebral cortex to plasma concentration at 2 hours after administration was 0.32 and 0.39, respectively. These findings suggest that GTWY peptide—or its metabolites—penetrate to the brain and directly improve memory function. Moreover, the hippocampal dopamine (DA) level was increased and the rate of DA metabolism decreased 1 hour after a single administration of GTWY peptide. GTWY peptide also inhibited monoamine oxidase B (MAO-B) activity. Treatment with dopamine D1 receptor antagonist SCH23390 attenuated memory improvement by GTWY peptide in the scopolamine-induced amnesia mouse model. These results indicate that the orally administered GTWY peptide is delivered to the brain where it directly inhibits MAO-B activity, resulting in the increased dopamine levels in the brain and in improved hippocampus-dependent memory function. Previous reports have indicated that DA is involved in the hippocampus-dependent memory function [5]. A dopamine precursor, levodopa (L-DOPA) has been shown to improve task-based learning rates and task performances in the elderly. A recent study revealed that sembragiline, a MAO-B selective inhibitor, has a preventive effect on neuronal loss and astrogliosis by reducing reactive oxygen species [6]. Taken together, WY-containing peptides from dairy products exert their beneficial effect on the prevention of age-related cognitive decline by increasing DA level in the brain.

We also found that GTWY peptide is included in various commercially available cheeses and yogurts, especially such as Brie, Stilton, and Camembert fermented by *Penicillium*. Whey peptides digested by certain enzymes were also revealed to contain abundant GTWY peptide. These support the results of the previous epidemiological investigations that the consumption of fermented dairy products prevented cognitive decline in the elderly. Our recent randomized, double-blind, placebo-controlled clinical trial demonstrated that supplementation with 1 g whey peptide rich in GTWY peptide for 6 weeks improved memory retrieval (verbal fluency test), attention, and executive function (Stroop test) in healthy adults, especially in those with cognitive fatigue [7].

In summary, peptides including WY were identified as responsible components in dairy products that improve memory function and age-related cognitive decline. The effective intake of WY-related peptides, such as the consumption of whey digestions, is beneficial for improving cognitive decline with age and ultimately leads to prevention of dementia.
